# Endometrial Injury and Its Rescue by Mesenchymal Stem Cells Is Dependent on Estrous Cycle Phase

**DOI:** 10.1111/jcmm.70966

**Published:** 2025-11-26

**Authors:** Ramanaiah Mamillapalli, Ying Liu, Yuping Zhou, Reshef Tal, Hugh S. Taylor

**Affiliations:** ^1^ Department of Obstetrics, Gynecology & Reproductive Sciences Yale School of Medicine New Haven Connecticut USA

**Keywords:** Asherman syndrome, bone marrow, endometrium, infertility, injury, mice, regenerative medicine, reproduction, stem cells, uterus

## Abstract

Asherman Syndrome (AS) is caused by injury to the endometrium leading to uterine scarring, decreased menstruation and infertility; it typically occurs after surgical curettage of the uterus. AS is treated surgically albeit with limited success. Administration of bone marrow‐derived mesenchymal stem cells (MSCs) has recently been demonstrated to restore uterine function in AS; however, there is no data available on the role of the estrous cycle phase on outcomes. Here, we describe endometrial injury during estrus or diestrus, its differential effect on fertility, and its response after bone marrow MSC treatment to reverse the infertility in a murine model. Endometrial injury in the estrus phase did not affect fertility outcomes whereas injury in the diestrus phase resulted in infertility. Bone marrow (BM)‐derived MSC treatment without injury in the estrus or diestrus phase did not affect the pregnancy outcomes. BM MSC treatment following endometrial injury in the diestrus phase restored fertility. Immunofluorescence analysis revealed that vimentin or cytokeratin‐positive BM‐derived cells in the uterus were extremely rare. BM MSC treatment after injury increased CD45^+^ cells, indicating a role for immunomodulation in endometrial repair. Finally, qRT‐PCR showed that *Ccl3*, *Il‐1β* and *Mmp3* gene expression was significantly higher in the endometrium of the injury + BM MSC group than in other groups. In summary, injury to the endometrium during the diestrus phase results in infertility that can be restored by the treatment of BM MSCs. The therapeutic effect of BM MSCs on the endometrium appears to be mediated primarily by immunomodulation rather than BM MSC engraftment.

## 1 Introduction

Infertility is highly prevalent worldwide, affecting 1 in 6 couples attempting to conceive [1, 2]. There are multiple etiologies of infertility; however, uterine trauma and subsequent injury are some of the most poorly understood and difficult to treat [3, 4]. Uterine injury can lead to intrauterine adhesions and synechia, resulting in decreased menstrual flow, amenorrhea, or infertility. Clinically, intrauterine scarring is termed Asherman syndrome (AS). This syndrome is most typically casually associated with instrumentation of the post‐partum uterus. The incidence is over 20% in patients undergoing procedures within the first month after pregnancy termination, missed abortion, or vaginal delivery. While the occurrence after pregnancy suggests a hormonal influence on susceptibility, there have been no studies done to identify if the phase of the estrus or menstrual cycle affects AS in the non‐pregnant woman. Surgical treatment is the current standard therapy for AS; however, this therapy is not always successful. No other treatments are clinically available or widely used. Recent evidence in animal models has demonstrated that uterine damage is improved by the administration of mesenchymal stem cells (MSCs) that promote endometrial repair and regeneration [5, 6]. Due to their proven tissue repair function and easy availability, MSCs are the most promising stem cells for tissue regeneration [7]. Bone marrow (BM)‐derived MSCs have been used in several studies specifically looking at endometrial regeneration [8, 9, 10].

Bone marrow‐derived cells (BMDCs) include MSCs have been shown to differentiate into multiple nonhematopoietic cell lineages in the uterus and to play a role in the reconstitution of the human endometrium [11]. Several endometrial cell populations were found to be derived from labelled donor bone marrow [12, 13, 14, 15]. BMDCs are recruited to the murine endometrium in response to ischemia/reperfusion injury [16] and are a normal part of pregnancy, including post‐partum repair [17, 18, 19].

In previous studies involving endometrial injury leading to AS, BM derived MSCs have been demonstrated to improve endometrial regeneration. BM transplantation in Asherman's syndrome mice can not only repair the uterus but also improve fertility [20]. Further, BMDCs transplant from wild‐type mice can induce endometrial regeneration and rescue pregnancy loss in mice harbouring a targeted mutation in Hoxa11, which has a uterine specific infertility defect [19]. In women with Asherman's or endometrial atrophy treatment with CD133^+^ bone marrow‐derived stem cells increased endometrial thickness and angiogenesis; 10 out of 16 patients with Asherman's syndrome and/or endometrial atrophy were pregnant spontaneously or after embryo transfer [21].

There has been no report of endometrial injury performed in different estrus cycle phases in animal models. Moreover, no studies have been carried out on the effects of endometrial injury followed by BMDCs treatment in these different phases of the cycle. The present study aimed to determine whether mice in the estrus or diestrus phases are more susceptible to endometrial damage and infertility following endometrial injury and if the success of BMDCs treatment also depends on the phase of the cycle. As part of developing novel clinical treatments to address endometrial injury, it is equally important to know the effects of reproductive cycle phase in susceptibility to uterine damage and for precise targeting of treatments.

## 2 Materials and Methods

### 2.1 Animals

Wild‐type C57BL/6J adult female virgin mice and fertile males were obtained from Charles River Laboratories (Wilmington, MA). Transgenic C57BL/6 L mice expressing enhanced green fluorescent protein (GFP) under regulation of the ubiquitin C (UBC) promoter were obtained from Jackson Laboratory and used as bone marrow donors. All animals were maintained in the Animal Facility of Yale University School of Medicine. Mice were housed in an animal room exposed to a 12 h light/dark cycle (7:00 am–7:00 pm) with food and water provided *ad libitum*. All animals were treated under an approved Yale University institutional animal care and use committee protocol.

### 2.2 Experimental Design

**FIGURE 1:**
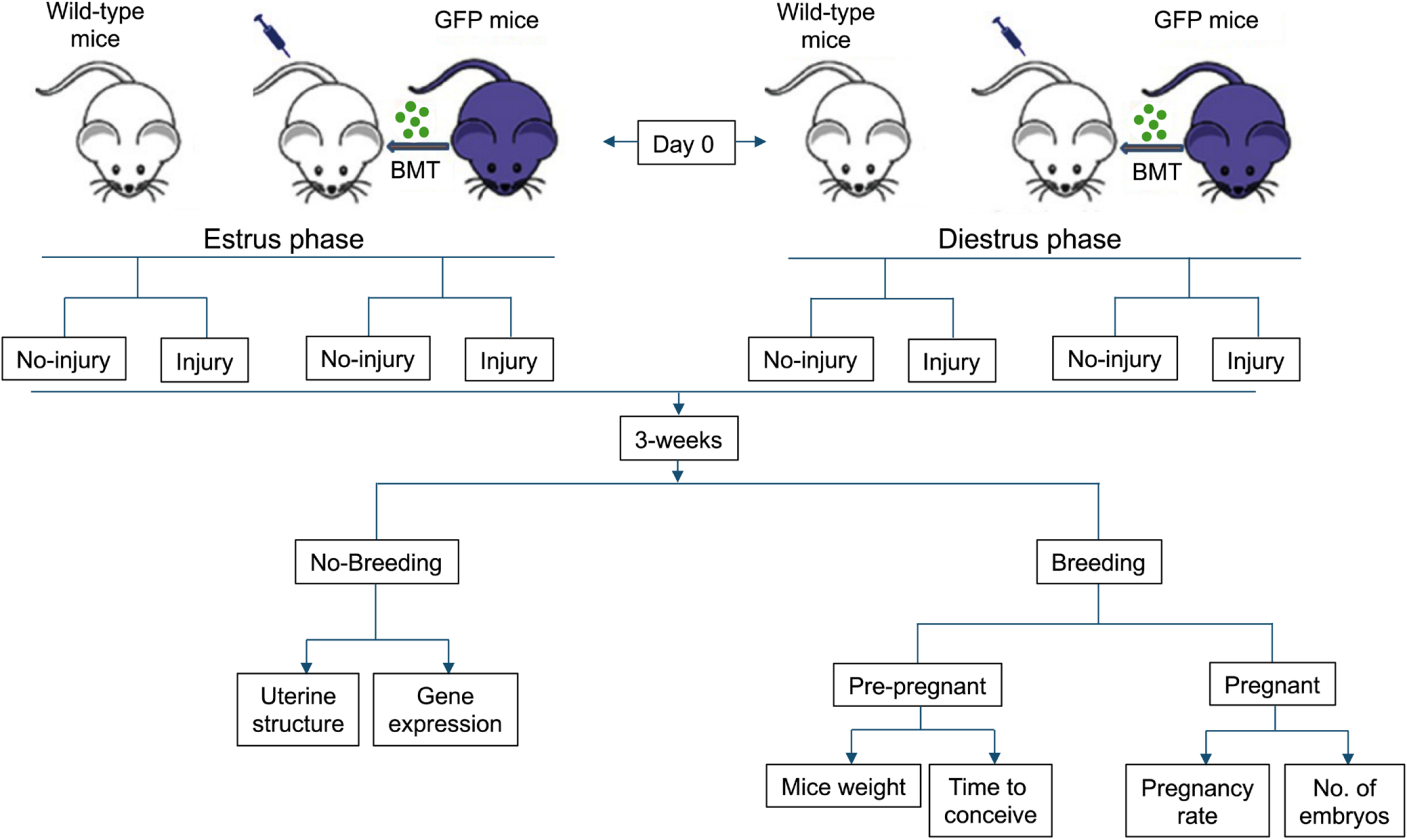
A schematic depicting the different regimens of systemic administration of BMDCs in mouse uterine injury models performed in estrus and diestrus phase. Mice underwent endometrial injury and received BMT (BM MSCs) as a treatment regimen. Mice were divided into four groups in each of the estrus and diestrus phases. Group 1: No endometrial injury. Group 2: Endometrial injury. Group III: No endometrial injury and BM MSC injection. Group IV: Injury + BMT group: Endometrial injury and BM MSC injection. Three weeks of post‐treatment with BM MSCs mice were bred, and fertility outcomes were monitored. Separately, non‐pregnant uterine tissues were subjected to immunofluorescence and quantitative RT‐PCR for GFP cells engraftment and gene expression, respectively. BM, Bone marrow; BMT, Bone marrow transplant; GFP, green fluorescent protein; MSC, Mesenchymal stem cell.

Six‐week‐old female C57BL/6J wild‐type mice were allocated into eight groups (*n* = 16 in each group), according to the estrus or diestrus phase identified by daily vaginal cytology and received BMT and/or endometrial injury treatment regimens as shown in Figure 1. Group I (no‐injury): estrus phase; no endometrial injury, had sham surgery by laparotomy alone; no BMT, received 100 μL saline by tail vein injection; serving as control. Group II (injury): estrus phase; received endometrial injury and 100 μL saline by tail vein injection. Group III (injury + BMT): estrus phase; received endometrial injury and 1 × 10^7^ BM MSCs in 100 μL saline by tail vein injection. Group IV (no‐injury + BMT): estrus phase; no endometrial injury, had sham surgery by laparotomy alone and received 1 × 10^7^ BM MSCs in 100 μL saline by tail vein injection. Group V (no‐injury): no endometrial injury, had sham surgery by laparotomy alone; no BMT, received 100 μL saline by tail vein injection; serving as control. Group VI (injury): diestrus phase; received endometrial injury and 100 μL saline by tail vein injection. Group VII (injury + BMT): diestrus phase; received endometrial injury and 1 × 10^7^ BM MSCs in 100 μL saline by tail vein injection. Group VIII (no‐injury + BMT): diestrus phase; no endometrial injury, had sham surgery by laparotomy alone and received 1 × 10^7^ BM MSCs in 100 μL saline by tail vein injection.

### 2.3 Vaginal Cytology

Vaginal cytology was performed by daily vaginal smear to assess estrus or diestrus cyclicity before the endometrial injury procedure. To obtain vaginal cells, a sterile swab was moistened with saline and rotated against the vaginal wall. Sequentially, the vaginal cells were smeared onto a slide, mounted with cover slips, and visualised under a light microscope. Similarly, vaginal cytology was also performed to assess estrus cyclicity 5 days after BMT and/or endometrial injury. These assessments indicated that mice in all treatment groups were undergoing normal estrus cycles after BMT injection and/or endometrial injury.

### 2.4 Endometrial Injury

Prior to BMT, 6‐week‐old female C57BL/6J wild‐type mice were subjected to mild uterine injury according to our prior protocol [17] with minor modification. Briefly, after administration of isoflurane (Isothesia; Henry Schein, Ohio), a vertical incision was made in the abdominal wall and the uterus was exposed under sterile conditions. A 27‐gauge needle was inserted into the lumen of each uterine horn at the utero‐tubal junction, rotated, and withdrawn ten times gently.

### 2.5 Bone Marrow Cells Isolation and Transplantation

Bone marrow cells were flushed from the femurs and tibias of 6–8‐week‐old C57BL/6‐Tg UBC‐GFP mice with sterile DMEM/F12 as described previously [17]. After that, the bone marrow cells were filtered using 70 μm mesh, centrifuged at 2000 rpm for 5 min, washed and resuspended with phosphate buffered saline. Recently, we found that the systemic route of administration of BM MSCs resulted in better recruitment to the injured uterus than local injection [17] hence, we used the intravenous route of administration here. A total of 1 × 10^7^ bone marrow cells were injected intravenously into female C57BL/6J wild‐type mice according to the various regimens described above at the time of abdominal surgery.

### 2.6 Reproductive Studies

The next estrus cycle after endometrial injury and BMT, two female mice were mated with one male C57BL/6J mouse of proven fertility and checked daily for signs of pregnancy as described previously [13]. The mice were set to breed for a period of 1 month, and the female weight was measured daily to monitor pregnancy development. After 1 month of breeding, any remaining females were separated from the male and continued to be observed for 3 more weeks to monitor for pregnancy. Pregnancy was determined by weight gain and other physical signs. The pregnant mice were separated from the male and one group euthanized 1 week later by CO^2^ inhalation followed by cervical dislocation. The uteri were photographed, and the total number of implantation sites was determined. Time to pregnancy was counted by the days from the first breeding day to the first day of pregnancy, determined retrospectively from the delivery date in those groups that completed pregnancy. Pregnancy rates were calculated as a percentage of the number of pregnant mice divided by the total number of mice in each group.

### 2.7 Tissue Collection and Histological Analysis

Those recipient mice euthanized at 3 weeks post injury and/or BM transplantation were sacrificed by CO^2^ inhalation followed by cervical dislocation, perfusion with saline to remove intravascular blood, and uterus extraction. For each mouse, one horn was fixed in 4% paraformaldehyde, embedded in paraffin, and cut into 5‐μm sections for H&E staining and immunofluorescence while the other horn was placed in RNAlater and frozen at −80°C for subsequent RNA extraction and qRT‐PCR.

### 2.8 H&E Staining, and Immunofluorescence

As described previously [17], uterine tissues for histological analysis were fixed in 4% paraformaldehyde for 16–24 h (overnight) at room temperature, dehydrated, and then embedded in paraffin. Five micrometer sections were cut and mounted on slides, deparaffinized in xylene, rehydrated in graded ethanol washes, and then stained with H&E for assessment of endometrial histology.

Immunofluorescence was used to perform colocalization studies of GFP^+^ cells with vimentin or cytokeratin. Moreover, immunofluorescence was used to assess CD45^+^ leukocytes and CD31^+^ endothelial cells in the uterus. Blocking was applied with 10% donkey serum (Sigma‐Aldrich, St Louis, MO) in PBS for 60 min at room temperature. After blocking, sections were incubated overnight at 4°C with either of the following primary antibodies: polyclonal goat anti‐GFP (1:1000), rat anti‐vimentin (1:300), rat anti‐cytokeratin (1:200), rat anti‐CD45 (1:300), and rabbit anti‐CD31 (1:200) (all from Abcam, Cambridge, MA). The following secondary antibodies were used: AlexaFluor 564‐conjugated donkey anti‐goat, AlexaFluor 488‐conjugated donkey anti‐rabbit, and AlexaFluor 488‐conjugated donkey anti‐rat (all diluted in 1:200) (Life Technologies, Carlsbad, CA) for 1 h at room temperature. Nuclear counterstaining was performed by incubating slides with 4,6‐diamidino‐2‐phenylindole (DAPI) (Vector Laboratories, Burlingame, CA). Negative controls excluding primary antibody were included in every staining. All the visualisations of the slides were done with a laser scanning confocal microscope (LSM 710, Zeiss, New York, NY) and the ZEN software (Carl Zeiss).

### 2.9 Image Capture and Cell Counting

As described previously [17], for quantitative analysis of CD45^+^and CD31^+^ cells in the uterus, three random sections of slides from each mouse were captured at 400× magnification. Regions of endometrium were randomly chosen, and full thickness was counted. Images were obtained by adequate excitation and emission filter sets: DAPI for nuclei, fluorescein isothiocyanate for GFP, rhodamine for vimentin, cytokeratin, CD45 and CD31. They were subsequently analysed using Image J software (version 1.32j) (National Institutes of Health, USA). The total number of DAPI^+^ cell nuclei was counted, and the numbers of CD45^+^ and CD31^+^ cells were then counted and expressed as percentages of the total cell nuclei counted per section. At least 1000 cells were counted per animal.

### 2.10 Quantitative Real‐Time Polymerase Chain Reaction (qRT‐PCR)

Total RNA was extracted from endometrial tissues with Trizol reagent (Ambion, Quantity, Grand Island, New York) according to the manufacturer's instructions. The complementary DNA (cDNA) templates for PCR analysis were synthesised from the total RNA following the instructions of the first‐strand cDNA synthesis kit (Fermentas, Toronto, Canada). Real‐time PCR was performed using SYBR Green PCR Master Mix Reagent (SYBR Premix Ex Taq kit, Cat. DRR041A; TaKaRa Clontech, Mountain View, California) and the LightCycler 480 SYBR Green I Master (Roche Diagnostics International Ltd). The specific primers used for genes *Ccl3*, *Il‐1β* and *Mmp3* are as follows: *Ccl3*, F: 5′‐CCAGGTGTCATTTTCCTGACT‐3′ and R: 5′‐ATGCAGGTGGCAGGAATGTT‐3′; *IL‐1β*, F: 5′‐TGCCACCTTTTGACAGTGATG‐3′ and R: 5′‐AAGGTCCACGGGAAAGACAC‐3′; *Mmp3*, F: 5′‐GTCCCTCTATGGAACTCCCAC‐3′ and R: 5′‐AGGGTGCTGACTGCATCAAA‐3′ (Sigma) (Takara). All samples were a 10 μL aliquot of each reaction mix and were transferred to a well of a MicroAmp optical 96‐well reaction plate (MX3000P; Stratagene, Santa Clara, California). The reaction conditions were as follows: 95°C for 10 min; 40 cycles of 95°C for 10 s, 58°C for 20 s, and 72°C for 15 s; and melting curve from 60°C to 95°C, increasing in increments of 0.5°C every 5 s. Reactions were run in duplicate using RNA samples from three independent experiments. Water was used as a negative control. The fold change in expression of each gene was calculated using the 2^−△△CT^ method with β‐actin as an internal control.

### 2.11 Statistical Analysis

Statistical analyses were performed using the GraphPad Prism 5 software (GraphPad, San Diego, California). Descriptive statistical analysis was performed initially to examine the distribution of data. Differences in the number of implantations and the expression of CD45 and CD31 protein were analysed using the unpaired *t*‐test. The differences in the expression levels of *Ccl3*, *Il‐1β*, and *Mmp3* mRNA were determined by the Mann–Whitney *U* test. A *p*‐value < 0.05 was considered statistically significant.

## 3 Results

### 3.1 Endometrial Injury Cause Infertility in Diestrus Phase

**FIGURE 2:**
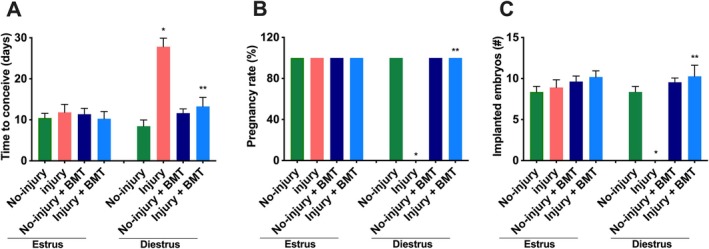
Reproductive outcomes following endometrial injury and/or BM MSCs injection. Mice in all four groups of each phase (estrus or diestrus) were mated with proven males and monitored daily for pregnancy for 1 month. (A) Time to conceive. In the diestrus phase injury group, the time to conceive increased significantly compared to the no‐injury group. BM MSC treatment reduced the time to conceive in the injury+BMT group compared to the injury alone group. Days of breeding were calculated from the first breeding day to the first pregnancy day extrapolated from the day of delivery. *p < 0.0002 vs. no‐injury and **p < 0.0005 vs. injury group. (B) Pregnancy rate. The pregnancy rate in the injury group in the diestrus phase was significantly reduced compared to the no‐injury group while the rate in the injury group with BM MSC (BMT) treatment was significantly increased compared to the injury group without treatment. The pregnancy rate was calculated as the percentage of pregnant mice divided by the total number of mice in its group. *p < 0.0001 vs. no‐injury and **p < 0.0001 vs. injury group. (C) Implanted sites or embryos. The number of embryos in the injury group in the diestrus phase was significantly reduced compared to the no‐injury group while the number in the injury group with BM MSC treatment was significantly increased compared to the injury group without treatment. *p < 0.0001 vs. no‐injury and **p < 0.001 vs. injury group. Data presented as mean ± SEM in (A) and (C) and percentages in (B).

To investigate the effects of endometrial injury on fertility, we induced endometrial injury in mice during the estrus as well as diestrus phases. As shown in Figure 2A, injury to the endometrium in the estrus phase did not have any significant effect on time to conceive (11.4 ± 3.6 vs. 10.3 ± 3.3 days, no‐injury vs. injured, respectively, *p* = 0.82). In the diestrus phase injury to the endometrium had a detrimental effect compared to the no‐injury group (> 30 vs. 9.0 ± 1.0, *p* = 0.0002). Similarly, time to conceive was significantly increased in the diestrus phase (> 30 vs. 11.4 ± 3.5, *p* = 0.0002) compared to the estrus phase in the injury groups (Figure 2A).

The pregnancy rate between the no‐injury and injured groups when injury was performed in the estrus phase was not significant (100% vs. 100%, *p* > 0.05) while the difference between the no‐injury and injured groups was significantly increased in the diestrus phase (100% vs. 0%, *p* < 0.0001; Figure 2B). No pregnancy was found after injury in the diestrus phase (0%) compared to the estrus phase (100%, *p* < 0.0001) as shown in Figure 2C. The numbers of viable embryos were similar between the no‐injury and injured groups in the estrus phase (8.3 ± 1.0 vs. 8.8 ± 0.9, *p* = 0.71) while in the diestrus phase no embryos were found in the injured group compared to the no‐injury group (0 ± 0 vs. 8.8 ± 0.9, *p* < 0.0001). The number of viable embryos was compared between the estrus and diestrus phases among injured groups; the number was significantly decreased in the diestrus phase group (8.8 ± 1.0 vs. 0 ± 0, *p* < 0.0001, Figure 2C).

### 3.2 BM MSCs Reverses the Endometrial Injury Effects on Fertility Outcomes

To determine the effect of BM MSCs on endometrial injury, we treated the mice with BM MSCs or controls, both in the estrus as well as in the diestrus phases, and analysed the fertility outcomes. In the estrus as well as the diestrus phases, BM MSCs treated groups without endometrial injury did not show any significant difference in time to conceive compared to the groups without treatment and injury (estrus: 10.3 ± 3.3 d vs. 10.3 ± 3.6 days, *p* > 0.999; diestrus: 9.0 ± 1.0 days vs. 16.0 ± 2.9 days, *p* = 0.13). Also, time to conceive was not significant between the two phases (estrus: 10.3 ± 3.3 vs. diestrus: 16.0 ± 2.9 days, *p* = 0.25) for the BM MSCs treated and untreated groups without injury (Figure 2A). Similarly, in the estrus phase the pregnancy rate in untreated and treated groups with BMDCs was 100% (Figure 2B). Also, we observed that there was no significant change in the number of viable embryos between treated and untreated groups without injury in both phases of estrus and diestrus as shown in Figure 2C (estrus: 8.3 ± 1.0 vs. 9.8 ± 0.4, *p* = 0.17; diestrus: 8.8 ± 0.9 vs. 9.8 ± 1.0 *p* = 0.48). There is no change in viable embryos between the estrus and diestrus phases for the same groups (Figure 2C, estrus: 9.8 ± 0.37 vs. diestrus: 9.80 ± 0.97, *p* > 0.99). These results showed no significant difference in time to pregnancy, pregnancy rates and number of viable embryos following BM MSC treatment compared to no treatment in the estrus phase of no‐injury groups.

However, BM MSCs markedly reversed the effects of endometrial injury in the diestrus phase. Time to conceive was reduced significantly in the injury group treated with BMDCs compared to the untreated injury group in the diestrus phase (13.8 ± 4.0 vs. 30 days, *p* < 0.05). There was no significant change between the two phases (estrus vs. diestrus) after BM MSCs treatment in the injured groups (11.0 ± 3.512 vs. 13.8 ± 4.0, *p* = 0.64). The pregnancy rates were 100% in all groups except in the untreated injury group which was 0% in the diestrus phase (*p* < 0.05) as shown in Figure 2B. The number of viable embryos was restored to normal levels in the injury group treated by BM MSCs compared to the untreated group without injury (9.3 ± 0.9 vs. 8.8 ± 0.9, *p* = 0.713), as shown in Figure 2C.

Taken together, these results reveal that endometrial injury in the estrus phase did not have any effect on fertility, either with or without treatment; however, injury to the endometrium in the diestrus phase had a significant detrimental effect. BM MSCs treatment restored fertility to normal levels.

### 3.3 Lack of Histologic Evidence of Fibrosis in the Mouse Uterine Injury Model

**FIGURE 3:**
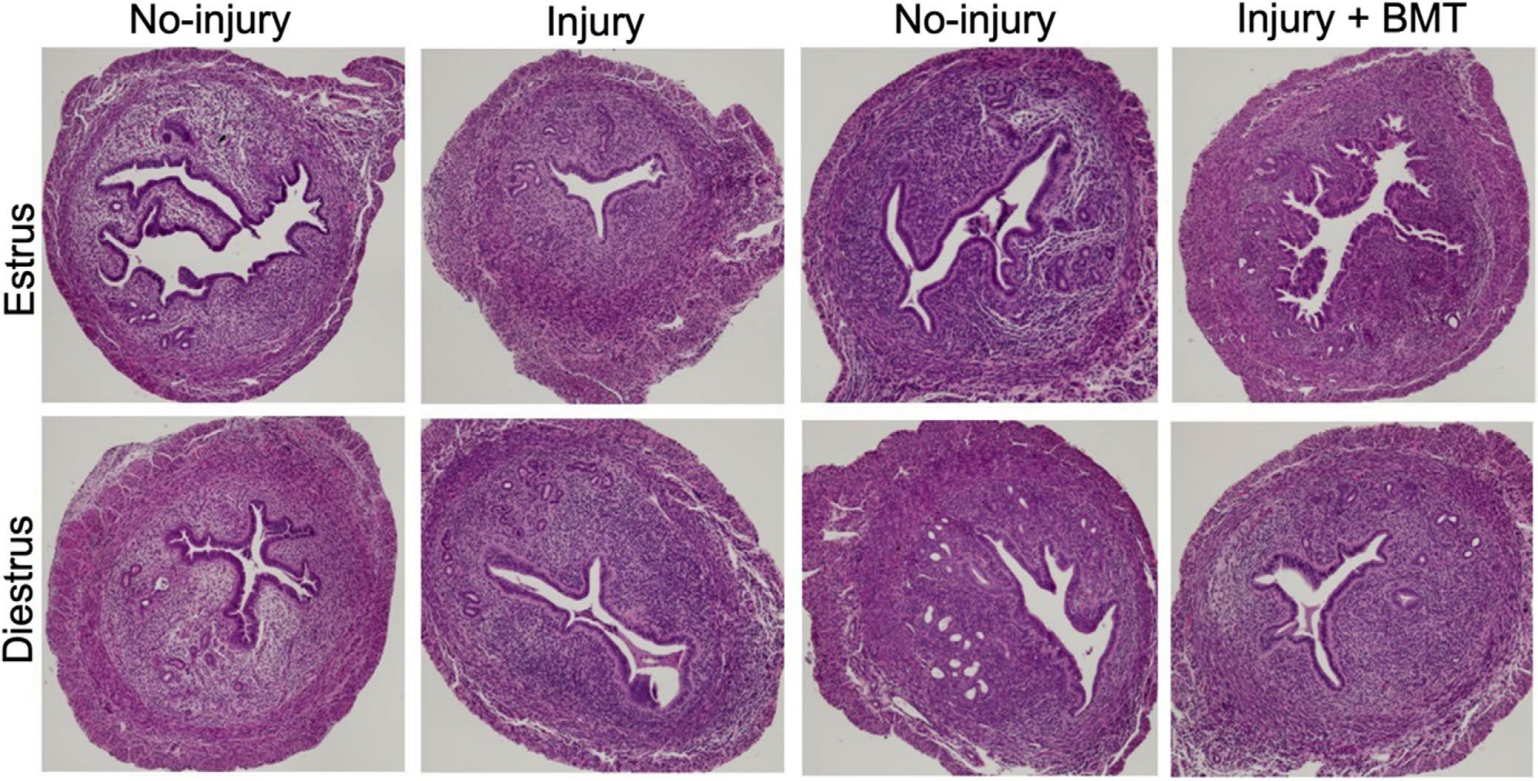
Representative images of uterus with H&E staining. Histological analysis was carried out on uteri 3 weeks after uterine injury in estrus or diestrus phases and/or BMDCs injection. Images from all groups from both estrus and diestrus phases showed normal endometrial histology without evidence of fibrosis or scarring. (Original magnification, ×100).

To evaluate whether the injured endometrium was repaired with or without fibrosis or scarring, uterine horns were collected for histological assessment at 3 weeks following endometrial injury and/or BMDCs injection. H&E staining of uterine sections for histological analysis is shown in Figure 3. Representative images showed that the injured endometrium, whether injury occurred in either the estrus or diestrus phase, had no obvious fibrosis or scarring. As expected, representative images from controls as well as BMDCs control groups showed that there was no significant histologic evidence of fibrosis. This reflects the relatively mild injury in this AS model.

### 3.4 BMDCs Repair the Endometrium Not by Differentiation Into Endometrial Cells

**FIGURE 4:**
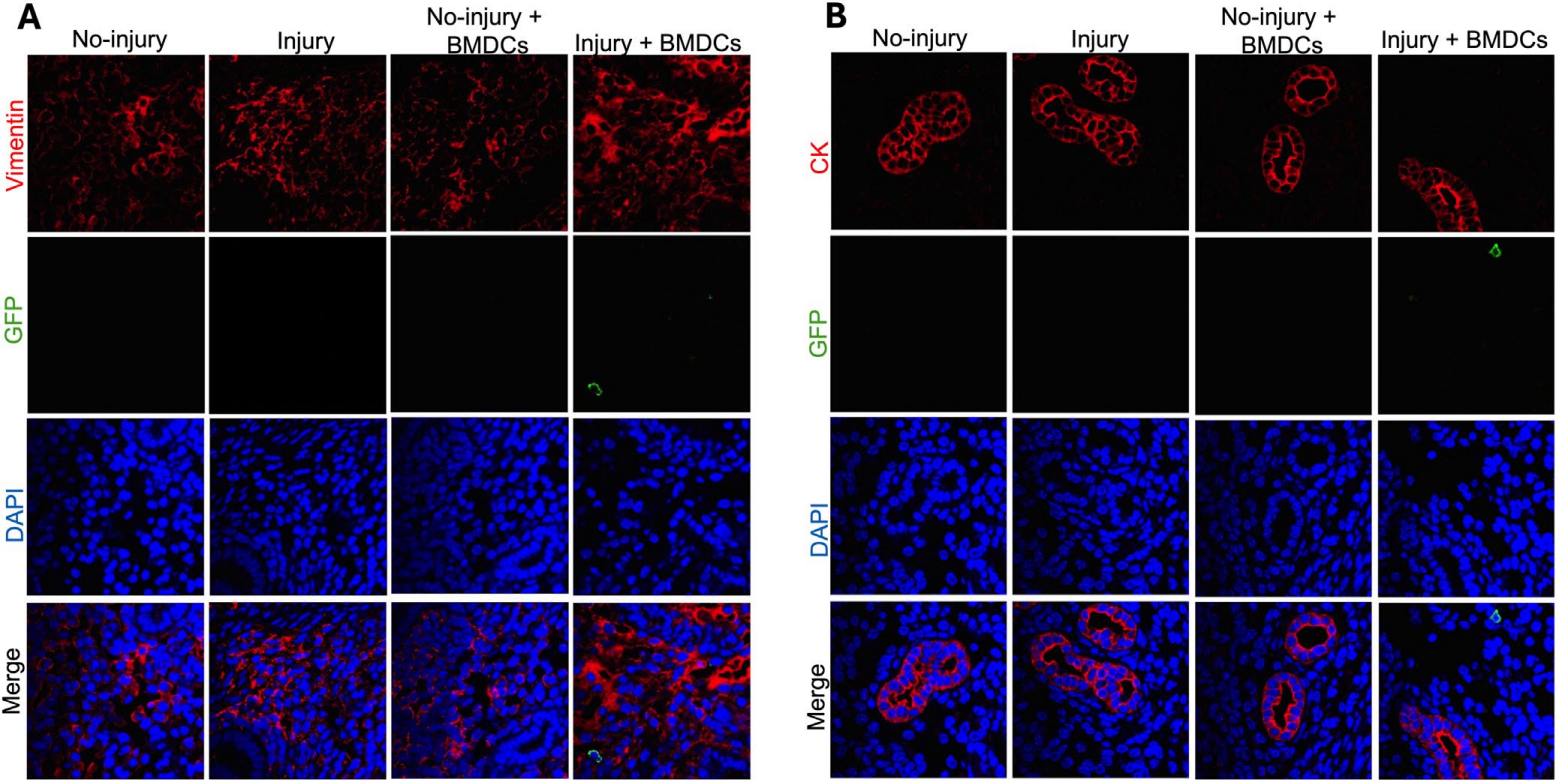
Representative images showing vimentin and cytokeratin by immunofluorescence staining. Uterine tissues were staining with anti‐GFP antibody (green) and co‐stained with either anti‐vimentin antibody, a stromal cell marker (red) (A), or with anti‐cytokeratin antibody, an epithelial cell marker (red) (B). GFP‐positive cells did not commonly colocalized with vimentin or cytokeratin.

Our previous study reported that the GFP^+^ cell percentage in the injured horn was only 0.22% at 3 weeks after BMDCs injection, suggesting that the number of BMDCs engrafted in the uterus was very low [17]. We next asked whether the recruited BMDCs differentiate into endometrial cells including stromal cells, epithelial cells, or other kinds of cells. The uterine sections were co‐stained using vimentin (stromal marker), cytokeratin (epithelial marker) antibodies to evaluate if they were co‐expressed with GFP^+^ cells. Consistent with our previous experiments [17], only rare GFP^+^ cells were found to be vimentin positive (data not shown). Typical results are shown in Figure 4A, where most GFP^+^ cells were not vimentin positive. Similarly, none were positive for both GFP and cytokeratin (Figure 4B). These results demonstrated that very few donor‐derived stem cells differentiated into endometrial stromal cells.

**FIGURE 5:**
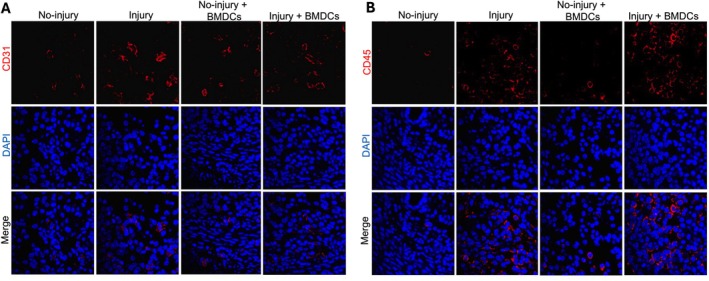
Representative images showing CD31 and CD45 by immunofluorescence staining. Uterine tissue sections were stained with anti‐CD31 antibody, an endothelial cell marker (A, red), and anti‐CD45 antibody for CD45, a pan‐leukocyte marker (B, red). Blood vessel density was not different in injury+BMT group compared with no‐injury group and no‐injury+BMT MSC group. All nuclei were stained by DAPI and are shown in blue (magnification ×400).

To investigate whether angiogenesis plays a role in the process of endometrium restoration, the horns of the injured mice were collected at 3 weeks after BMDCs injection, and angiogenesis was evaluated by staining for endothelial cells using CD31. The results showed that there was no increase in endothelial progenitor cells in the injured group or injured + BM MSC‐treated group compared with the no‐injury group or no‐injury and BM MSC‐treated groups (Figure 5A). Taken together, these data suggested that the BMDCs repair the endometrium/restore the fertility not through increased angiogenesis or by stem cell trans‐differentiation and clonal expansion.

### 3.5 BMDCs Repair the Endometrium and Through Immune Modulation

In addition, to test whether endometrial injury recruited immune cells to the uterine injury and if BM MSCs modified that response, the uterine horns were assessed for immune cells using the CD45 pan‐leukocyte marker. There were few CD45^+^ cells in the endometrium in no‐injury groups (with or without BM MSC‐treatment). The CD45^+^ cells were increased in the injury group and constituted approximately 5% of total cells (Figure 5B). After BM MSC treatment, the number of engrafted CD45^+^ cells nearly doubled. These data suggest that the BM MSCs repair the endometrium through immunomodulation of the response induced by injury.

**FIGURE 6:**
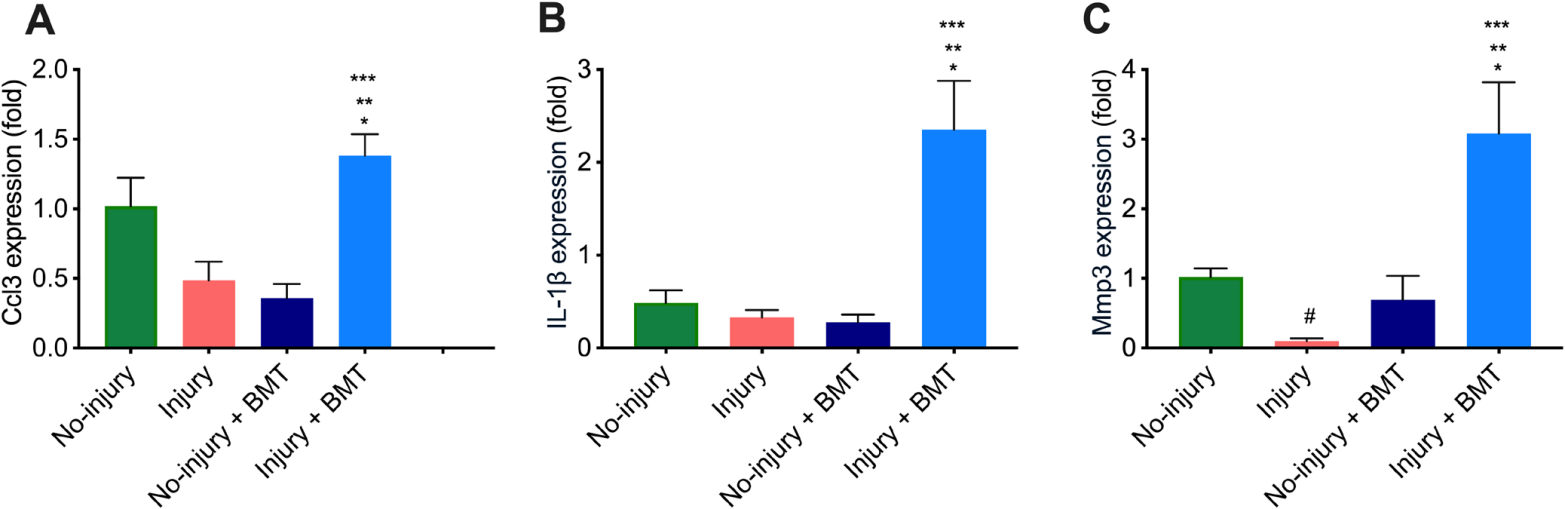
Expression levels of *Ccl3*, *Il‐1β*, and *Mmp3* by qRT‐PCR. Significant increases in the mRNA levels of *Ccl3* (A), *Il‐1β* (B), and *Mmp3* (C) in the injured + BMT group compared to the other groups. Each bar represents the mean ± SEM for data from three individual experiments, and each experiment was performed in duplicate. *p < 0.05 vs. no‐injury, **p < 0.05 vs. injury, and ***p < 0.05 vs. no‐injury + BMT. (A–C). ^**#**^ *p* < 0.05 vs. no‐injury (C).

In order to identify the mechanism by which BM MSCs repaired the injured endometrium in the diestrus phase, we identified several genes related to inflammation and immune response. Of these, gene *Ccl3*, shown in Figure 6A, was more highly expressed in the injury + BM group compared to the no‐injury group (*p* = 0.04), the injury alone group (*p* = 0.0006), or the no‐injury+BMT group (*p* < 0.0001). The mRNA expression levels of *Il‐1β* are shown in Figure 6B. *Il‐1β* was more highly expressed in the injury+BM group compared to the no‐injury group (*p* = 0.02), the injury group (*p* = 0.01) and the no‐injury group (*p* < 0.008). No significant differences were found among the other three groups (no‐injury, injury and no‐injury+BMT) for both *Ccl3* and *Il‐1β* expression levels.

*Mmp3* mRNA levels, as shown in Figure 6C, were significantly increased in the uterus of the injury+BMT group compared to the no‐injury group (*p* = 0.002), the injury group (*p* = 0.0009), and the no‐injury+BMT group (*p* = 0.0002). Moreover, a significant decrease in *Mmp3* mRNA levels was observed in the injury group compared to the no‐injury group (*p* = 0.0002).

The significant increase in the expression levels of *Ccl3*, *Il‐1β* and *Mmp3* in the injured uterus of mice that received BM MSCs during the diestrus phase suggested that inflammation‐mediated immune modulation may play an important role in the bone marrow‐mediated restoration of fertility after injury in the endometrium in the diestrus phase.

## 4 Discussion

No prior studies examining endometrial injury compared susceptibility and treatment in the different phases of the estrus cycle. Here we showed that controlled injury in the estrus phase has no effect on subsequent fertility or pregnancy. In contrast, injury in the diestrus phase results in infertility in mice. Here we used the same mouse injury model as previously developed in our laboratory, which consists of a mild endometrial injury that reduces infertility without forming uterine adhesions [17]. More severe injury may be detrimental in any phase.

Mice do not menstruate, and the mouse estrus cycle has distinct differences from the human menstrual cycle; despite these limitations, the estrus phase may be regarded as corresponding to the proliferative phase, and the diestrus phase to the secretory phase. If we extrapolate from this mouse study, it appears that injury in the proliferative phase may be more favourable than in the luteal phase. While the timing of endometrial surgical procedures during the menstrual cycle and the relationship to endometrial injury need further study in humans, these data would suggest that risks may be lower if surgery is performed in the proliferative phase. Previous reports of improved endometrial receptivity after mild injury or ‘scratching’ of the endometrium in the secretory phase have been disproven in large, randomised trials [22]. Oestrogen drives cell proliferation as well as stem cell recruitment to the endometrium [23, 24]. Rapid cell division in the proliferative phase may allow tissue regeneration while terminal decidualization/differentiation present in the secretory phase could impede the ability to regenerate after injury. Further, hormonal therapies used to control endometrial disorders such as endometriosis also influence stem cell migration and engraftment [25, 26]. Similarly, tobacco exposure limits endometrial regeneration and stem cell recruitment [27]. Hormonal levels as well as exposure to toxins should be considered in procedures that involve the endometrium. These data imply limiting progestin exposure and assuring sufficient oestrogen to limit endometrial injury and facilitate repair.

We previously reported that the percentage of donor GFP^+^ cells engrafted in the uterus was relatively very low: 0.22% at 3 weeks after BM MSC injection [17, 28]. Rare donor‐derived GFP^+^ cells were vimentin positive, but cytokeratin and CD31 negative, demonstrating that some donor‐derived stem cells differentiate into endometrial stromal cells but not epithelial cells or vascular endometrial cells [17]. The results of this study agree with the rare trans‐differentiation of stromal cells from BM MSCs. Together these results indicate that the repair induced by BM MSCs is likely indirect by secretion of local factors to stimulate the surrounding endometrial cells to proliferate actively to repair the injured endometrium, rather than direct differentiation into endometrial cells such as stromal cells or epithelial cells.

We have previously reported that uterine ischemia/reperfusion injury results in increased BM MSC recruitment to the endometrium of mice mediated by CXCL12 [16, 28, 29]. In that study, however, endometrial damage was induced by ischemia/reperfusion injury and not local mechanical injury, suggesting different mechanisms of stem cell‐mediated repair in different types of injury. In another study, we demonstrated that BM MSC transplantation improved fertility in a severe AS mouse model, where greater numbers of stem cells were identified in the endometrium. In that study injury to the endometrium was more substantial, leading to scarring and diminished fertility, unlike the mild endometrial injury used in the current study [20]. Asherman's syndrome is associated with infertility due to severe damage to the endometrial basalis layer. In contrast, local endometrial injury induces a mild wound that does not result in loss of the endometrial basalis layer. Both hormone levels, specifically of oestrogen and progesterone, as well as the degree of injury affect stem cell recruitment and recovery from injury.

In our present study, we found that CD45^+^ cells were significantly more abundant in the injury + MSCs group compared with the MSCs injection group or the endometrial injury alone group. This was consistent with other studies, which reported that monocytes were recruited to sites of injury [30]. Dekel et al. demonstrated that the implantation success resulted from the inflammatory reaction induced by trauma [31]. Gnainsky et al. showed that the expression of proinflammatory cytokines was upregulated by endometrial injury, recruiting monocyte/macrophages to the site of injury [32]. Here our results indicate that, not only does inflammation caused by injury recruit leukocytes, but the BM MSCs may also play a crucial role in the recruitment of leukocytes to the endometrium. We have previously shown that exposure to stem cells influences the local inflammatory milieu by releasing exosomes [12, 33]. This may be one mechanism by which BM MSCs influence leukocyte numbers in repairing local endometrial damage. Stem cell exposure also modifies leukocyte production and function by an effect on the bone marrow itself [34]. Not only is the local tissue affected by BM MSCs, but the bone marrow itself may also be remodelled, allowing for improved endometrial repair. Here we demonstrate that BM MSCs have an immunomodulatory role in endometrial repairs.

To further investigate the possible mechanism by which BMDCs regenerate endometrial receptivity following injury in the diestrus phase, we analysed the uterine gene expression as related to immune mechanisms in the four diestrus mouse groups. We showed that three genes *Ccl3*, *Il‐1β*, and *Mmp3* were more highly expressed after endometrial injury and BM MSC treatment. CCL3 (also known as macrophage inflammatory protein 1 alpha, MIP‐α) belongs to the CC chemokine family. It plays a role in inflammatory responses involved in the acute inflammatory recruitment and activation of inflammatory cells. Interleukin‐1β (IL‐1B) is a member of the interleukin 1 family of cytokines. This proinflammatory cytokine, secreted by immune cells including macrophages and neutrophils, is involved in multiple events related to activating and regulating inflammation [35, 36]. Matrix metalloproteinases (MMPs) are a family of calcium and zinc‐dependent extracellular matrix (ECM) degrading enzymes, involved in physiological processes such as tissue remodelling as well as pathological processes including wound repair and angiogenesis in inflamed tissue [37, 38, 39, 40]. Overexpression of MMPs, especially MMP3, may facilitate the process of invasion and tissue remodelling [41].

The macrophage‐attracting chemokine may account for the increased leukocyte infiltration in the injury+BM MSC treated mice. Previous studies have shown that CCL3 is expressed in multiple tissues after injury. In accordance with those reports, our results demonstrated CCL3 expression was significantly upregulated in injured endometrium after BM treatment, but not in injured endometrium without BM treatment or just BM MSCs injection without injury. Based on these data, we postulate that BM MSCs regulate the secretion of CCL3, thus enhancing the infiltration and activation of immune cells such as macrophages and neutrophils. These invading immune cells produce proinflammatory cytokines such as IL‐1β, resulting in the secretion of MMP3. MMPs in turn degrade fibronectin, laminin, collagens and cartilage proteoglycans as well as promote stromal cell and epithelial cells to proliferate, overall leading to the repair of the wounded endometrium. Taken together, the physiological function of CCL3 and IL‐1β‐induced MMP3 might play a crucial role in endometrial remodelling by BM MSCs.

## 5 Conclusions

In summary, endometrial injury in the estrus phase does not affect fertility, but in the diestrus phase has a detrimental effect resulting in infertility in mice. Administration of bone marrow‐derived mesenchymal stem cells can restore fertility after injury in the diestrus phase. Bone marrow‐derived stem cells repair endometrial injury not by repopulating with new endometrial cells through trans‐differentiation. Rather, regeneration likely occurs through immunomodulation.

## Author Contributions

**Ramanaiah Mamillapalli:** conceptualization (equal), formal analysis (equal), investigation (equal), methodology (equal), project administration (equal), supervision (equal), writing – review and editing (equal). **Ying Liu:** data curation (equal). **Yuping Zhou:** methodology (equal). **Reshef Tal:** methodology (equal). **Hugh S. Taylor:** conceptualization (equal), funding acquisition (equal), writing – review and editing (equal).

## Funding

This work was supported by Clinical Center, R01 HD076422 and HD052668.

## Conflicts of Interest

The authors declare no conflicts of interest.

## Data Availability

The data that support the above findings are available from the corresponding author.
